# *Strophanthus sarmentosus* Extracts and the Strophanthus Cardenolide Ouabain Inhibit Snake Venom Proteases from *Echis ocellatus*

**DOI:** 10.3390/molecules30122625

**Published:** 2025-06-17

**Authors:** Julius Abiola, Olapeju Aiyelaagbe, Akindele Adeyi, Babafemi Ajisebiola, Simone König

**Affiliations:** 1 IZKF Core Unit Proteomics, University of Münster, 48149 Münster, Germany; 2 Organic Unit, Department of Chemistry, University of Ibadan, Ibadan 200005, Nigeria; 3 Animal Physiology Unit, Department of Zoology, University of Ibadan, Ibadan 200005, Nigeria; 4 Department of Animal and Environmental Biology, Osun State University, Osogbo 230001, Nigeria

**Keywords:** viper, cardenolides, metalloproteinase, plant extract, medicinal plant, snakebite

## Abstract

*Strophanthus sarmentosus* is recognised for various ethnomedicinal applications, including treatment after snakebites. However, only limited scientific evidence exists on its antivenomous capabilities. This study investigates the efficacy of methanol and ethylacetate extracts from *S. sarmentosus* leaves and roots against *Echis ocellatus* venom. A non-toxic range for the extracts was determined in rats, and assays were performed to test their anti-hemorrhagic and anti-hemolytic activity as well as their influence on venom-induced blood clotting. In all of these experiments, the extracts demonstrated significant positive effects equal to or better than antivenom. Moreover, the extracts strongly inhibited and even abolished the digestion of the vasoactive neuropeptide bradykinin by snake venom metalloproteinases. Strophantus plants are known for their high content of cardiac glycosides, one of which is the commercially available ouabain, that by itself also considerably inhibited venom-induced bradykinin cleavage. Although ouabain is only present in low amounts in *S. sarmentosus* when compared to other cardenolides of similar structure, it can be hypothesized that members of this substance class may also have inhibitory properties against venom proteases. *S. sarmentosus* additionally contains bioactive substances such as flavonoids, terpenoids, tannins, saponins, and alkaloids, which contribute to its protective effects. The study provides scientific data to explain the success of the traditional use of *S. sarmentosus* plant extracts as a first aid against envenomation in rural Africa.

## 1. Introduction

Snakebites pose a significant public health threat with potentially life-threatening consequences. Globally, approximately 5.4 million snakebite incidents occur annually, leading to 1.8 to 2.7 million cases of envenomation and an estimated 81,410 to 137,880 fatalities in addition to countless cases of permanent disabilities. In sub-Saharan Africa alone, there are around 300,000 reported snakebite cases each year, causing approximately 32,000 deaths. Many survivors are left with severe local tissue damage and chronic disabilities [1,2,3,4,5].

The *Viperidae* family is particularly notorious for human envenomations [6]. Vipers are distributed across Africa and Saudi Arabia, exhibiting significant intrageneric genetic diversity and low levels of monophyly [7]. This genetic variability among viper species contributes to a wide range of venom compositions, complicating treatment protocols and antivenom efficacy [8]. Diet and prey preference influence venom adaptation, making venoms more effective against specific prey [9]. Additionally, geographical location plays a role, with environmental factors driving regional variations in venom composition. Venom gland regeneration can also lead to slight compositional changes as the gland replenishes after a bite [10]. Sexual dimorphism may cause differences in venom toxicity and enzymatic activity between males and females in certain species [9,10].

*Echis ocellatus*, commonly known as the carpet viper, is recognized for its potent venom, which comprises a multitude of substances (for review on snake venom composition, see [11]). VenomZone [12] gives an overview of the venom components determined with both proteomic and transcriptomic analyses and illustrates the high abundance of snake venom metalloproteinases (SVMPs). Among the toxic proteins [13], SVMPs [14] and phospholipases A2 (PLA2s) [15] are the primary enzymes responsible for the deleterious effects observed following envenomation. SVMPs are generally associated with hemorrhage, the proteolytic degradation of fibrinogen and fibrin, the induction of apoptosis, and the inhibition of platelet aggregation during snakebite, and they are responsible for the pathological envenomation phenotypes caused particularly by species from the *Viperidae* family and the *Crotalinae* subfamily [14]. They contribute to the degradation of collagen and other structural proteins within the extracellular matrix, while PLA2s specifically target skeletal muscle and cardiac tissues, inducing necrosis in these areas. The action of these enzymes on the cardiovascular system is particularly concerning, as they can interfere with hemostatic mechanisms, including blood clotting and thrombosis, ultimately resulting in significant tissue damage [16]. The disruption of coagulation pathways can lead to uncontrolled bleeding or pathological clot formation, exacerbating vascular injury and impairing normal circulatory function [5].

The primary treatment for snakebites remains the administration of antivenom, which is derived from antibodies purified from the plasma of herbivorous animals (e.g., horses, donkeys, sheep) following their immunization with venom [17,18,19]. However, the use of antivenom has limitations [20]. One significant issue is the inadequacy of effective serum immunoglobulins, which can result in suboptimal therapeutic outcomes. Additionally, clinical side effects associated with antivenom administration can pose serious drawbacks in managing snakebite envenoming, particularly in rural communities where access to healthcare is limited. In sub-Saharan Africa, the challenges surrounding antivenom use are particularly pronounced [21]. The scarcity of available antivenom, coupled with high costs, creates significant barriers to treatment. Moreover, the preservation of antivenom is complicated by the frequent lack of electricity in rural areas. Thus, there is an urgent need for alternative treatments and strategies to address the public health crisis posed by snakebites in these regions.

Historically, herbal remedies have been utilized in traditional medicine to treat snakebite wounds effectively [22]. In Nigeria, various ethnomedicinal plants are employed for the immediate treatment of snakebites, among which *Strophanthus sarmentosus* stands out for its potent therapeutic properties, including anti-arthritis, antipyretic, emetic, anti-rheumatic, and diuretic effects [23,24]. Despite its traditional usage, the antivenomous potential of *S. sarmentosus* has yet to be rigorously scientifically validated.

In an earlier work, we demonstrated broad-spectrum activities against both gram- positive and gram-negative bacteria and fungi caused by *S. sarmentosus* extracts and showed the presence of tannins, saponins, glycosides, flavonoids, phenols, steroids, terpenoids, and carbohydrates [24] (for more information on the composition, see also recent review [23]). Some of these substances exhibit antioxidant, antimicrobial, antidiarrheal, anthelmintic, antiallergic, antispasmodic, and antiviral activities [25] and may have potential as protease inhibitors. The present study investigates the inhibitory potential of leaf and root extracts of this plant against the toxic effects induced by the venom of *Echis ocellatus*. Moreover, we use pure ouabain, the only commercially available cardiac glycoside of the Strophanthus species, as a surrogate substance and test its inhibitory activity.

## 2. Results

**Figure 1:**
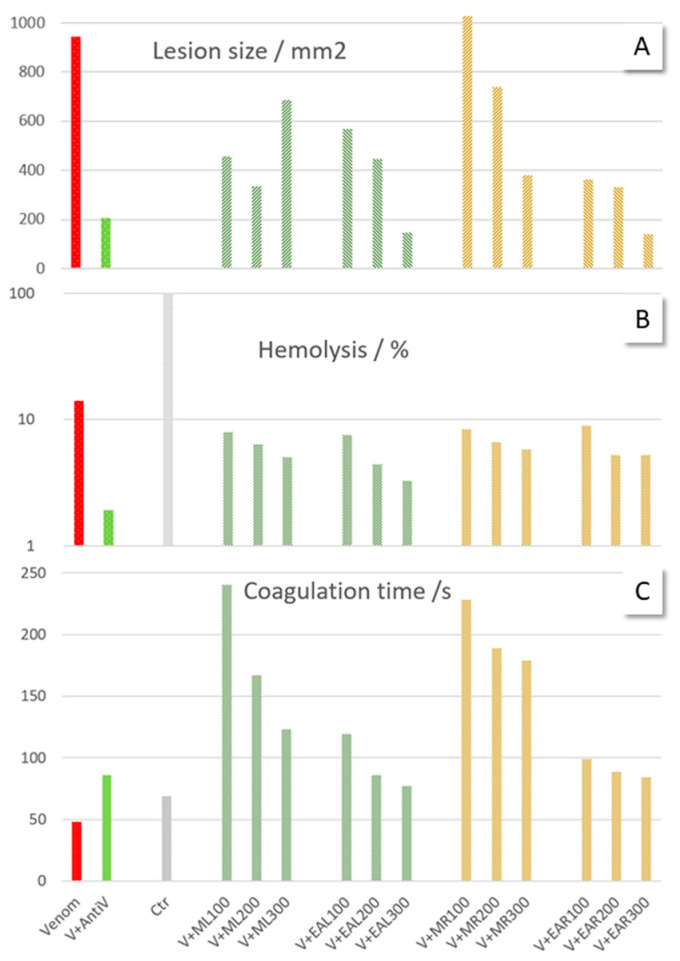
Summary of hemorrhage, hemolysis, and coagulation experiments to demonstrate the anti-venomous effects (V—venom, AntiV—antivenom; Ctr—control) of *S. sarmentosus* leaves (L) and roots (R), methanol (M), and ethylacetate (EA) with extracts at different concentrations (mean values). (**A**) Anti-hemorrhagic activity as expressed in the size of lesions on rat skin (100, 200, 300 mg/kg). (**B**) Anti-hemolysis activity. (**C**) Average blood coagulation time. (**B**,**C**) 100, 200, 300 mg/mL. For data, see Supplementary Tables S1–S5.

All data are given below and in Supplementary Tables S1–S5. Figure 1 serves as a visualisation of the hemorrhage, hemolysis, and coagulation experiments using plant extracts of different concentrations. For details on the experimental set-up, see Section 4, Materials and Methods.

### 2.1. Determination of Lethal Dose (LD_50_) of S. sarmentosus Extracts

No cases of morbidity or mortality were observed in rats within 48 h following the administration of varying concentrations of the methanol bulk extract redissolved in saline (175, 525, 1575, 4725 mg/kg body weight), suggesting that the extracts caused no acute toxicity or disruption of normal physiological functions. The animals remained active and showed no signs of physiological distress or intoxication for up to 96 h, indicating that the tested doses for *S. sarmentosus* extracts were well below the lethal dose 50% threshold (LD_50_). They animals exhibited normal fur condition with no piloerection (air ruffling), skin inflammation, or hair loss. Feeding and water intake remained consistent, indicating stable appetite and hydration. No abnormal behavior such as restlessness, lethargy, or hyperactivity were noted. The extract doses used in this study thus fell within the safe range for biological testing.

### 2.2. Anti-Hemorrhagic Activity of S. sarmentosus Extracts

**Figure 2:**
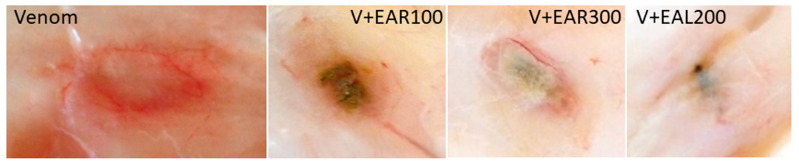
Hemorrhages on rat skin as induced by venom and venom (V) + ethylacetate (EA) extracts from roots (R) and leaves (L) at different concentrations (100, 200, 300 mg/kg). Example images, not to size. For data, see Table 1, Figure 2, and Supplementary Table S1.

Snake venom is known to induce hemorrhage primarily via the proteolytic activity of SVMPs, which degrade extracellular matrix components and disrupt vascular integrity [26]. To assess this effect, we intradermally injected venom into the skin of rats and evaluated the resulting hemorrhagic lesions. No hemorrhagic skin lesions were observed in the saline control group of rats, while venom injections caused the appearance of roundish-reddish areas of about 4 cm in diameter (Figure 2). The co-administration of leaf and root extracts with the venom led to a significant reduction in lesion size, confirming their inhibitory potential. Injections with venom + extracts lead to smaller lesions of greenish-to-black colouring. The determination of the lesion size was subjective due to variances in shape and frayed borders, so we calculated the area by measuring length and width and assuming an elliptic form (Figure 1A, Table 1, Supplementary Table S1). Treatment with antivenom significantly inhibited venom-induced hemorrhage, reducing both the red colour’s intensity and the size of the afflicted skin area. Methanol and ethylacetate extracts of *S. sarmentosus* generally showed a dose-dependent inhibition of hemorrhagic activity, with leaf and root ethylacetate extracts at the highest concentrations (300 mg/kg) achieving even better effects than antivenom. Methanol root extracts at low concentrations were not very effective, in contrast to those of ethylacetate. However, none of the treatments achieved a complete suppression of hemorrhage.

### 2.3. Anti-Hemolytic Activity of S. sarmentosus Extracts

The venom-induced hemolysis of erythrocytes is predominantly attributed to the enzymatic activity of PLA2, which hydrolyzes phospholipids in cell membranes, leading to membrane disruption and red blood cell lysis [27]. In our study, the venom-induced hemolysis of bovine erythrocytes (Figure 1B, Supplementary Table S2) was effectively countered by antivenom. *S. sarmentosus* extracts demonstrated a dose-dependent reduction in hemolysis. Higher extract concentrations yielded stronger protective effects against blood cell lysis; in the case of leaf ethylacetate extract, it reduced hemolysis to approximately 23%. Blood samples treated with high doses of extract (300 mg/kg) showed the most pronounced inhibition, with half-maximal inhibitory concentration (IC_50_) values ranging from 104 to 189 mg/mL (Table 2, Supplementary Table S3). Pure ouabain (0.5, 1, 2 mg/mL) exhibited only a moderate dose-dependent ability to preserve red blood cell (RBC) integrity, with an IC_50_ value of 3 mg/mL (Supplementary Table S4).

### 2.4. Coagulation Experiments

Snake venom contains anticoagulant proteins that significantly modulate hemostatic processes by targeting various coagulation factors and platelet functions [28]. In our experiments, venom administration led to a one-third reduction in coagulation time compared to citrated blood samples, an effect that was reversed upon the addition of antivenom. Notably, all plant extracts exhibited anticoagulant activity, with methanol extracts at the lowest tested dose prolonging clotting time up to fivefold compared to the venom effect.

Higher extract concentrations prevented coagulation for durations comparable to that of antivenom, suggesting a dose-dependent inhibitory effect on the clotting cascade. In citrated blood samples, clotting occurred at 69 s in the normal control group; with venom, the blood clotted faster (48 s, Figure 1C, Supplementary Table S5). Antivenom prevented clotting for 85 s. The addition of *S. sarmentosus* extracts counteracted the venom-induced hypercoagulation by shifting the onset of clotting to 77–240 s. Methanol leaf and root extracts at the lowest dose (100 mg/kg) were the most effective. The observed clotting time for pure ouabain (116 s) indicated a prolonged coagulation process compared to the hypercoagulation induced by the venom (Supplementary Table S5).

### 2.5. Cleavage of Dabsylated Bradykinin by Snake Venom

**Figure 3:**
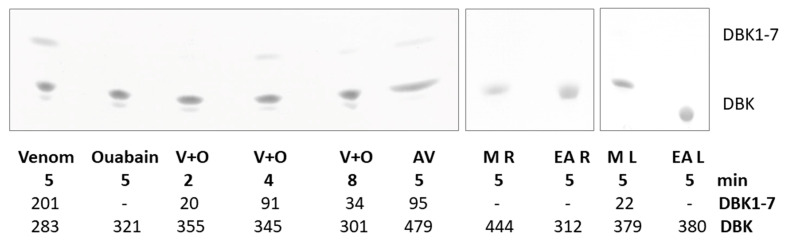
TLC separation of the products of the incubation of DBK with venom and varying additives (plant extracts and ouabain, respectively). The incubation time and the detected spot volumes are given (arbitrary units of colour density as determined by JustTLC software). Venom (no inhibitor), ouabain (no venom), V + O (venom + ouabain), AV (antivenom). *S. sarmentosus* extracts: M R/L (methanol extracts of root/leaf), EA R/L (ethylacetate extracts of root/leaf). The DBK spot was sometimes split into a major and a minor spot for unknown reasons.

We reported the degradation of dabsylated bradykinin (DBK) by *E. ocellatus* venom earlier [29]. In a 5 min incubation with venom, about half of the DBK was cleaved. The separation of the mixture resulted in two spots on a thin-layer chromatography (TLC)-plate, one for residual original peptide and one for fragment DBK1-7 (Figure 3). This activity was inhibited by antivenom so that only ~20% was digested to produce DBK1-7. Both root and leaf ethylacetate extracts, as well as the root methanol extract, completely abolished DBK cleavage. Only for the methanol leaf extract did some residual activity remain (~6% product). When ouabain was used as a surrogate substance for the cardenolides in *S. sarmentosus* and added to DBK before the addition of venom, DBK degradation was also considerably compromised. After 2 min of incubation, only 6% product was formed; after 8 min, it was 11%.

## 3. Discussion

### 3.1. Phytochemical Screening

While the cardiac glycosides of Strophanthus are well-researched natural compounds [23,30], detailed scientific investigations on other substance classes and their biological activities are still scarce. In an earlier phytochemical analysis of methanol, hexane, and ethylacetate extracts from the stem, leaves and roots of *S. sarmentosus* (for details, see Table 2 in [24]), we detected tannins, saponins, glycosides, flavonoids, phenolics, steroids, terpenoids, and carbohydrates. Phenolics, tannins, and alkaloids were particularly abundant in root methanol extracts, but all of these substance classes except for the terpenoids were found. In root ethylacetate extracts, no signals for flavonoids, saponins, and glycosides were seen, while in leaves, glycosides were indicated. Methanol extracted saponins, carbohydrates, glycosides, alkaloids, and steroids from leaves.

Here, we continued these efforts with a number of activity tests of plant extracts against *E. ocellatus* venom, working with non-toxic doses of methanol and ethylacetate leaf and root extracts (100–300 mg/kg) in comparison to the use of antivenom. It is important to note that we did not collect data concerning the long-term toxicity of the extracts beyond 96 h, but traditional ethnomedical practice confirms safe use. Extrapolating from the work of other authors, extracts administered at 175 and 525 mg/kg may potentially pose low to moderate long-term toxicity risks, primarily causing mild organ stress. Doses of 1575 and 4725 mg/kg could induce severe acute toxicity, possibly resulting in chronic organ damage or genotoxicity in surviving animals. The chemical composition of the extract, including potential hepatotoxic or nephrotoxic constituents, plays a critical role for long-term effects. Additionally, factors such as bioaccumulation and species-specific metabolism influence toxicity outcomes [31].

Pharmacokinetically, these doses result in limited gastrointestinal absorption of some of the bioactive compounds such as cardiac glycosides, which are known for their low to moderate bioavailability (see Section 3.3). These glycosides are efficiently metabolized by hepatic cytochrome P450 enzymes and rapidly excreted via urine and bile, preventing toxic accumulation even at 4725 mg/kg. Cardiac glycosides inhibit Na^+^/K^+^-ATPase (NKA), potentially increasing cardiac contractility, but the absence of significant effects across all doses suggests subthreshold inhibition as observed in acute toxicity studies [31,32].

### 3.2. Hemorrhage, Hemolysis, and Coagulation

Snake venom is known to cause hemorrhage primarily due to SVMP activity [26]. We investigated this effect with injections into rats’ skin. The use of leaf and root extracts together with venom reduced lesion size, confirming their inhibitory influence, particularly at the highest tested concentration. Notably, none of the treatments achieved a complete inhibition of hemorrhage, which can be due to several factors including the incomplete neutralization of toxins, individual variability, irreversible vascular damage, and interference from other biological molecules [26,33].

Hemolysis induced by venom on erythrocytes has been attributed primarily to PLA2 activity [34]. In our experiments, the anti-hemolytic activity of the plant extracts did not equal that of antivenom but was, nevertheless, remarkable and reduced hemolysis to ~23% when leaf ethylacetate extract was used.

Anticoagulant proteins in snake venom significantly influence hemostatic processes [35]. In our study, venom reduced the coagulation time in comparison to citrated blood samples by about a third, a process which was resolved by the addition of antivenom. The use of all types of extracts extended the onset of clotting up to five-fold for the lowest dose of methanol extracts. Higher doses prevented coagulation for about as long as antivenom.

The difference in biological activity between methanol and ethylacetate extracts arises from their respective polarities, which determine the types of bioactive compounds each solvent can extract. Ethylacetate, a medium-polarity solvent, predominantly extracts moderately polar or lipophilic compounds such as flavonoid aglycones, terpenoids, and certain alkaloids, while methanol is a highly polar solvent and extracts hydrophilic compounds like phenolics, tannins, and glycosides [36,37]. Ethylacetate extracts directly influence membrane stabilization, specific coagulation pathway inhibition, and clot formation, demonstrating superior anti-hemorrhagic, anti-hemolytic, and anticoagulant effects, whereas methanol extracts primarily offer generalized antioxidant effects in aqueous environments and capillary strengthening properties [38,39,40,41].

Bioactive compounds in plant extracts with anti-hemorrhagic activity support hemostasis by promoting platelet aggregation, enhancing fibrin synthesis or stabilization, and reinforcing vascular walls, thereby reducing capillary fragility, improving vascular repair and strength, and minimizing blood loss [42]. Flavonoid aglycones, lipophilic terpenoids, moderately polar phenolics, and tocopherols (vitamin E) are effective in such biological processes involving lipid-rich environments like cell membranes [38,43,44]. They are known to inhibit coagulation enzymes or platelet aggregation and modulate the clotting cascade by interacting with lipid-binding sites or enzymes like thrombin and fibrinogen [38,39,43]. Lipophilic compounds effectively penetrate biological membranes, enhancing anti-hemolytic and anticoagulant activities [38,45,46,47]. Moderately polar compounds from ethylacetate extracts interact efficiently with enzymes and proteins, improving coagulation and RBC activation and stabilization [39,43]. The higher presence of specific enzyme inhibitors in these extracts makes them more effective at targeting and modulating specific coagulation pathways [39,48,49]. Lipophilic compounds (e.g., tocopherols and nonpolar flavonoids) integrate into RBC membranes, protecting them from oxidative stress, stabilizing the lipid bilayer, and preventing hemolysis [45,50,51,52]. The lipophilic nature of these compounds makes ethylacetate extracts more effective at stabilizing RBC membranes compared to more polar extracts [53]. The synergistic action of diverse bioactive compounds further strengthens the effectiveness of ethylacetate extracts compared to methanol extracts [39,43].

Hydrophilic compounds like flavonoid glycosides, tannins, and phenolics, while bioactive, are less bioavailable and less effective in these pathways [54,55,56]. They play a significant role in inhibiting platelet aggregation by interacting with receptors on platelet surfaces. They also chelate metal ions, such as calcium, disrupting coagulation cascades. Additionally, these compounds exhibit general anti-inflammatory effects, which may further modulate clot formation [38,45,57].

Anti-hemolytic substances protect RBCs from rupture by scavenging reactive oxygen species to prevent oxidative damage, but high polarity makes them less efficient at directly stabilizing lipid-rich RBC membranes [58,59]. Tannins from methanol extracts form protein–tannin complexes, which strengthen vascular walls and decrease capillary permeability [49].

Plant extracts showed better effects in some tests than antivenom, which is still the treatment of choice after snakebite. The mechanisms of action are different: while antivenom acts based on antibody–antigen binding, the complex chemical composition of plant extracts may include, other bioactive compounds besides enzyme inhibitors, enabling a variety of potentially synergistic processes, which are mostly unknown. Antivenoms contain antibodies which effectively recognize and neutralize one or more specific antigens and are limited in their application to certain species. Plant extracts, on the other hand, due to their heterogeneity containing a multitude of bioactive substances, may provide valuable broad-band initial treatment [60].

### 3.3. Inhibition of Venom Enzymes by Cardiac Glycosides

In addition to demonstrating the protective properties of *S. sarmentosus* extracts against hemorrhage, hemolysis, and disturbances of the coagulation cascade, we can show the inhibition of venom metalloproteinases using the neuropeptide BK as a reporter substance (for method, see [61]). BK is vasoactive and a substrate of angiotensin-converting enzyme (ACE), the inhibitors of which are commonly used in the treatment of hypertension [62,63]. Earlier, in unrelated research, we have used DBK to monitor the serum activity of ACE and basic carboxypeptidases N and B2 (CPN, CPB2) in two clinical studies on Complex-Regional Pain Syndrome and COVID-19 [64,65,66]. Interestingly, DBK was quickly cleaved by *E. ocellatus* venom enzymes at position 7-8, generating fragments of DBK1-7 in a few minutes [29]. This activity was almost completely abolished by the addition of EDTA, indicating SVMP activity. Antivenom considerably reduced DBK cleavage, while the extracts completely abolished it. Only for the methanol leaf extract did some residual activity remain (~6% product).

Venom protease digestion of DBK seems to generate only this one product (BK sequence: RPPGFSP↓FR) in contrast to proteases of human serum or blood, where DBK1-5 and DBK1-8 are the major fragments [61]. A new scan of the MEROPS database (www.ebi.ac.uk/merops, accessed 21 August 2024; for earlier discussion of protease specificity, see [29]) indicated the following peptidases, which could cleave the sequence motif GFSPFR at this position: peptidyl-dipeptidases Acer, Ance, and Dcp, ACE, thimet oligopeptidase, neurolysin, tropolysin, coccolysin, matrix metallopeptidase 8, lebetase, neprilysin and neprilysin-2, endothelin-converting enzymes 1 and 2, insulysin, plinsulysin, dactylysin, prolyl oligopeptidase, and, interestingly, HT-1 peptidase from *Crotalus viridis* and BmooMPα-1 from *Bothrops* sp. The latter are both SVMPs, and it is likely that similar activity is also present in *E. ocellatus* venom. Candidate proteins, which we detected in *E. ocellatus,* were zinc metalloproteinase-disintegrin-like Eoc1 (Uniprot accession Q2UXR0) and disintegrin EO4A (Q3BER3) [29]; the Uniprot database hosts 53 entries for SVMPs in *E. ocellatus,* with only small differences in sequences.

In our earlier work [29], we also noted residual activity which did not seem to originate from SVMPs. Serine protease fragments D5KRX9 and D5KRY1 of *E. ocellatus* were detected, as well as hints for serine protease B5U6Y3. It will, however, be necessary to properly separate and isolate individual proteins and validate their sequences before further conclusions on their specific activity can be drawn.

Given the fact that *S. sarmentosus* has been traditionally used to treat snakebite, especially in Nigeria [22,23], and is a source of potent cardenolides, we hypothesized that its cardiotoxins may have a role in venom protease inhibition. Therefore, we tested pure ouabain for its enzyme-inhibitory activity. Indeed, inhibition by ouabain was more effective than that by antivenom, indicating that the cardenolide class of substances could considerably contribute to the inhibitory properties of *S. sarmentosus* extracts. Of course, the modes of inhibition are different: antivenom acts based on antibody–antigen binding, an interaction between large protein molecules, while ouabain is a small molecule of 585 Da that apparently can not only bind to the sodium–potassium pump NKA [30,67] but also to phosphatases [68] and metalloproteinases [29].

While ouabain, like other cardiac glycosides, acts on the carrier system [69,70], its ability to preserve erythrocyte membrane integrity was only moderate in our hemolysis experiments, suggesting a low therapeutic potential compared to *S. sarmentosus* extracts. Plant-derived compounds, including cardiac glycosides, may influence cell membrane stability [71]. While some glycosides are associated with hemolysis, others, particularly those found in medicinal plants, may exhibit protective effects by reinforcing membrane structure and mitigating oxidative or mechanical stress on erythrocytes.

However, ouabain seems to play a role in blood coagulation, potentially by modulating clotting factor activity. The mean clotting time of 116 s indicated a prolonged coagulation process compared to the hypercoagulation induced by *E. ocellatus* venom, which significantly accelerated clotting to 48 s. Thus, ouabain did not enhance rapid clot formation but instead exerted a stabilizing effect on blood coagulation or possessed mild anticoagulant properties [72]. This result aligned with the findings for *S. sarmentosus* extracts, where higher concentrations delayed clotting times up to 240 s, implying the presence of bioactive compounds that interact with coagulation pathways. Apparently, cardenolides such as ouabain can contribute to that effect.

Historically, ouabain was mainly isolated from *S. gratus* seeds [23]. In *S. sarmentosus*, only traces have been detected, but it contains a mixture of similar glycosides of the sarmentogenin/saverogenin class, which have been elucidated in detail by the Reichstein group [73]. Their toxicity is based on the inhibition of NKA, which disrupts ionic homeostasis, leading to elevated Ca^2+^ concentration and cell death [30,67]. Ouabain is relatively highly soluble in aqueous media, in contrast to other cardenolides, and is thus a favoured inhibitor in identifying NKA and studying its physiological effects [74]. A 2024 review on ouabain and NKA summarizes their impact on physiology and pathophysiology in several points [74]: (1) Subnanomolar ouabain concentration may stimulate NKA, while higher concentrations are inhibitory. (2) Endogenous ouabain and other cardiotonic steroids were discovered in mammalian circulation, including humans. (3) NKA isoforms differ in structure and sensitivity to cardiotoxins. (4) The NKA is both a cation pump and a hormone receptor/signal transducer. Ouabain binding to NKA activates protein kinases and Ca^2+^-dependent signaling cascades with widespread physiological effects that can contribute to hypertension and heart failure. (5) Cardiotonic steroids differ in their effects depending on their individual structures.

Regarding snakes, ouabain increased the rate of creatine kinase release by *Bothrops jararacussu* crude venom in isolated mouse muscles [75]. Moderate rat brain NKA inactivation by PLA2 from *Naja naja oxiana* venom was accompanied by a decrease in the affinity for ouabain [76]. Myotoxin α (not a protein) from *Crotalus viridis viridis* venom reduced the resting membrane potential of mouse and rat diaphragms, and the depolarizing effect was augmented by ouabain [77]. Notably, ouabain was shown to inhibit both venom ATPase and phosphatase in *Bitis gabonica*, *B*. *arietans*, and *E. carinatus* [68].

In a computational study, to examine the inhibition of SVMPs by phytochemicals present in *Andrographis paniculata*, an annual herbaceous plant in the family *Acanthaceae* native to India and Sri Lanka, ouabain showed the highest similarity score with the phytochemical andropanoside, which is known to possess a protective activity against various liver disorders [78]. These authors were mainly interested in the SVMPs from Russell viper venom.

## 4. Materials and Methods

### 4.1. Animals

All animal experimental procedures were conducted in accordance with approval from the University of Ibadan’s Animal Care and Research Ethics Committee (ACUREC/005-0122/17:05/12/2022A) and in agreement with ARRIVE guideline 2.0. The study followed the National Code for Health Research Ethics guidelines. Additionally, all experiments adhered to the National Institutes of Health’s Guide for the Care and Use of Laboratory Animals, 8th edition, 2011. This study used the pooled venom of 13 adult male snakes of *E. ocellatus* from the wild in Kaltungo, Nigeria, as described before [29]. The antivenom was a gift from EchiTAB-Plus-ICP, Instituto Clodomiro Picado, Universidad De Costa Rica, San José, Costa Rica. The EchiTAB PLU-ICP polyvalent antivenom serum was stored at 4 °C until use. Male albino Wistar rats were acquired from the Animal Facility of the Department of Physiology, College of Medicine, University of Ibadan, Ibadan, Nigeria, acclimatized for two weeks, and fed ad libitum.

### 4.2. Preparation of S. sarmentosus Extracts

Plants were collected from the premises of Forestry Research Institute, Ibadan, Oyo State, Southwest, Nigeria (7°39′11″ N, 3°85′82″ E). The plant samples were identified and authenticated by D. P. O. Esimekhuai at the Herbarium of the Botany Department, University of Ibadan, and a voucher specimen (UIH 23178) of the plant was deposited at the herbarium for further reference. The air-dried plant material (2 kg of each leaf and root) was ground using a hammer mill with a 5 KVA motor (Grinding machine, Emmytex inv. Nig. Limited, Lagos, Nigeria). It was then extracted by maceration at room temperature (37 °C) in 50% aqueous methanol for 72 h and subsequently filtered (Whatman Quantitative Filter Paper, Cytiva, Buckinghamshire, UK; 10.5 s, 100 mL flow rate, level 1, 42.5 mm diameter). This 50% aqueous methanol bulk extract was further fractionated using liquid–liquid partitioning, applying first n-hexane and then ethylacetate. After the solvents were removed using a rotary evaporator (Buchi, Merck, Darmstadt, Germany), the dry extracts were stored in amber-coloured bottles in the refrigerator. Extracts were redissolved in NaCl solution (9 g/L) for use. Hexane extracts proved to be not as effective in some inhibition experiments and were thus not further studied.

### 4.3. Determination of Lethal Dose (LD_50_) of Plant Extract

LD_50_ of the methanol extracts of *S. sarmentosus* were determined as described before, with few modifications [79]. In accordance with the OECD guidelines for the testing of extracts or molecules (OECD Test Guideline 425), animals were grouped based on similar body weights to reduce variability and ensure consistent dosing. The administered dosages were calculated based on the mean body weight of each group to achieve accurate dose delivery per kilogram of body mass, as recommended by the OECD for weight-adjusted dosing. This ensured that each animal received a dose appropriate for its body mass, thereby minimizing inter-group variability and enhancing the accuracy of the toxicological evaluation. Twelve groups of rats (*n* = 5, 100–135 g) were given an oral administration of 175, 525, 1575, and 4725 mg/kg body weight of the 50% aqueous methanol root and leaf extracts redissolved in saline [80], respectively, while five rats were saline-injected and used as a control. The animals were monitored closely for up to 72 h and checked upon for up to 96 h.

### 4.4. Hemorrhage Assay

The antihemorrhagic activity of *S. sarmentosus* roots and leaves were measured as described, with slight modifications [81,82]. Rats (145–185 g) were randomly divided into six groups (*n* = 3) for treatment with 0.1 mL saline (control), venom, venom + antivenom (0.1 mL), or venom + 100/200/300 mg/kg body weight extracts (methanol and ethylacetate, 0.1 mL), respectively. Rats were envenomed by a single intradermal injection on their back of 0.1 mL of LD_50_ (0.22 mg/mL) of venom dissolved in 1 mL saline. The extract + venom and venom + antivenom (0.2 mL) mixtures were incubated at 37 °C for 1 h and subsequently injected into the animal. After 3 h, rats were euthanised, the skin was peeled away, and the size of the hemorrhagic lesion at the injection site (length, width, cross-section) was determined using a vernier caliper. The measurements were subjective due to variances in shape and frayed borders. We thus estimated the lesion area by assuming an elliptic form. We have intentionally chosen this method for its clarity over using the so-called percentage hemorrhagic inhibition value, which is also calculated based on the mean lesion dimensions (length, width, and cross-section), but averages them first across all samples [81,82].

### 4.5. Hemolysis Assay

This assay was performed as described before, with 20 mL of citrated bovine blood obtained fresh from a local butcher in a citrated tube [81,83]. Erythrocytes were washed 10 times with 5 mL of saline (0.9%) and centrifuged (2.400 rpm, 10 min). The RBC pellet was resuspended to a 10% (*v*/*v*) suspension in saline. Venom (0.2 mL of 0.22 mg dissolved in 1 mL of saline, LD_50_) was mixed with 2 mL of the cell suspension and 0.2 mL of different concentrations of *S. sarmentosus* extracts (100, 200, 300 mg/mL) or antivenom in separate tubes. The mixtures were incubated at 37 °C for 60 min. The reaction was terminated by adding 3 mL of chilled phosphate-buffered saline (PBS, pH 7.2). Tubes were centrifuged at 2.400 rpm for 10 min, and the absorbance of the supernatant was taken at 540 nm (Dual-beam Optical System, Perkin Elmer, Waltham, MA, USA). For the 100% hemolysis control, RBCs (2 mL of the 10% cell suspension) were treated with 3 mL distilled chilled water, causing cell lysis. Pure ouabain (Sigma, St. Louis, MO, USA) was tested at concentrations 0.5, 1, and 2 mg/mL (0.2 mL) using Ultrospec 2000 (Pharmacia, Sofia, Bulgaria) in the same manner, but in a new set of experiments. 

### 4.6. Coagulation Assay

Coagulant activity was measured using citrated bovine plasma [81,83]. Test samples were prepared in PBS to maintain physiological pH and ensure sample stability. For the assay, 0.2 mL of each sample—consisting of 0.2 mL of plasma mixed with 0.2 mL of PBS in a 1:1 ratio—was used for analysis. Plasma incubated with PBS was the control, while in the neutralization experiments, a constant amount of venom (0.2 mL of 1.0 mg venom in 1 mL of PBS) was reacted with various dilutions of *S. sarmentosus* extracts (0.2 mL of 100, 200, and 300 mg/mL in saline) or antivenom (0.2 mL), respectively. For the experiments with pure ouabain, a fixed volume of venom (0.2 mL of 1.0 mg/mL venom solution in PBS) was mixed with 0.2 mL of ouabain at varying concentrations (0.5, 1.0, and 2.0 mg/mL in saline). Mixtures were incubated for 30 min at 37 °C. Then, 0.2 mL of each mixture was added to 0.2 mL of citrated plasma containing 0.2 mg/mL calcium chloride. The clotting time was determined by manual observation and timekeeping. In control tubes, plasma was incubated with either venom or plant extracts.

### 4.7. DBK Degradation Assay

The experiments were performed as described [61] with slight changes [29]. Briefly, venom (3 µL, 0.2 mg/mL) was added to 350 pmol of dried DBK and incubated at 37 °C for 5 min or other time periods when specifically mentioned. Ouabain (Merck, Darmstadt, Germany; in 5% acetonitrile containing 0.1% formic acid) and extracts (reconstituted in 10% aqueous dimethylsulfoxide), respectively, were added (1.5 µL, 2 mg/mL) to DBK and incubated before the addition of venom (1.5 µL, 0.2 mg/mL in H_2_O). The controls (ouabain + DBK without venom; solvent, DBK + venom; venom + DBK; venom, antivenom + DBK) were processed in the same way. The reaction was quenched with ice-cold acetone and subsequent freezing of the sample. The supernatant obtained after centrifugation was dried and resuspended in 1.5 µL MeOH for TLC. The mobile phase was a mixture of CHCl_3_/CH_3_OH/H_2_O/CH_3_COOH (11:4:0.6:0.09 *v*/*v*/*v*/*v*). TLC sheets were scanned using a conventional flatbed scanner (Canon IJ Scan Utility, ScanGear v20.0.40, Krefeld, Germany) and analyzed with JustTLC v4.0.3 (Sweday, Sodra Sandby, Sweden).

### 4.8. Data Analysis

Data are presented as mean ± standard error. One way Analysis of Variance (ANOVA) was used to compare significant (*p* < 0.05) differences among groups. All statistical analyses were performed using Statistical Package for Social Sciences (SPSS) version 16.0 and GraphPad Prism 7.0.

## 5. Limitations

This study is limited by a number of factors in addition to the experimental windows set by the methods which were used. First, venom was obtained from snakes caught in the wild and may thus have been subject to geographical, nutrition, or seasonal influences (see Introduction). Second, the plants were grown in the open and were influenced by environmental conditions. Third, we were working with plant extracts of only roughly defined composition, so specific modes of pharmacological action could not be described at this stage. Fourth, the long-term toxicity of plant extracts beyond 96 h was not investigated. Fifth, a time interval typically elapses between a snakebite and the administration of first aid. This parameter was not considered.

## 6. Conclusions

In Nigeria, the use of medicinal plants, including species of the *Strophanthus* genus, is deeply woven into everyday life, from treating common illnesses to supporting cultural practices (spiritual cleansing rituals, protective charms), especially in rural and traditional communities. Extracts from *Strophanthus* roots, bark, leaves, and seeds are traditionally used to manage serious health conditions, including malaria, fever, snakebites, inflammation, and pain relief. The unique cardiotonic effects of these plants arise from their cardiac glycosides, which improve heart function when used in controlled doses. Due to their anti-inflammatory and antimicrobial actions, *Strophanthus* extracts are effective antivenoms and wound-healing agents and are helpful as first aid in rural settings.

This study demonstrates that methanol and ethylacetate extracts from the leaves and roots of *S. sarmentosus* exhibit notable antivenomous properties at non-toxic concentrations, effectively neutralizing pathological effects induced by *E. ocellatus* venom. Stem samples have also been tested in preliminary experiments but were not very active in comparison. The findings provide some scientific explanations for the successful traditional use of *S. sarmentosus* in snakebite treatment.

We are now progressing to the analysis of extract subfractions in the hunt for the individual active components because the biological effects cannot be confidently assigned to specific compounds without further fractionation and chemical validation. We could already show that the pure Strophantus cardenolide ouabain is a strong inhibitor of (metallo)proteases of *E. ocellatus* venom and has anti-coagulation properties. Although ouabain was only detected in minor quantities in *S. sarmentosus* plants, about 20 sarmentogenin/saverogenin glycosides have been described in the species, which vary only slightly in steroid core and sugar chains; it can be expected that some of them contribute to the activity.

The specificity of ouabain for venom-induced DBK cleavage is highly interesting because it enables a new line of venom research. The fact that venom proteases may target the vasoactive neuropeptide BK in human victims is of great interest, as it is a key factor in blood pressure homeostasis and part of inflammatory signalling networks.

Furthermore, the DBK-based assay is a fascinating tool to test plant extracts for active compounds. Its use may be more rewarding than the assays based on artificial peptide substrates typically used in venom studies.

In summary, simple alcohol extracts of *S. sarmentosus* plant parts significantly alleviate the effects of envenomation. The traditional ethnomedicinal use of the plant has demonstrated its safe application, and commercial products of this plant have been marketed before. Thus, further commercial development of Strophanthus extracts for human use does not seem too far away. However, given the history of the plant as a poison, it is still necessary to determine safe dosage and composition for specific applications. Proper chemical analysis and validation of individual bioactive candidates is, however, time-consuming. The use of ouabain, which is already available on the market as a certified reference material for research purposes, may be an interesting target as an adjunctive to antivenom use, but it also requires careful clinical testing to balance the cardioactive and antivenomous properties of the compound.

## Figures and Tables

**Table 1:** Anti-hemorrhagic activity of *S. sarmentosus* leaf and root extracts as expressed in the size of lesions on rat skin. Rats were injected with 0.2 mL of either saline, venom, venom + antivenom, or venom + extracts. Data are expressed as means ± StD of three individual experiments. For all measurements, see Supplementary Table S1.

	Extract/mg/kg	Lesion Area/mm^2^				
			Leaf		Root	
			Methanol	Ethylacetate	Methanol	Ethylacetate
**Venom**		943				
**Antivenom**		205				
**Saline**		No foci				
**Venom**	**100**		457	569	1026	363
	**200**		334	446	737	333
	**300**		687	148	380	141

**Table 2:** Effect of *S. sarmentosus* leaf and root extracts on hemolysis activity of venom (IC_50_, mg/mL).

Extract	Methanol	Ethylacetate
**Leaf**	161.90	104.45
**Root**	188.89	166.70

## Data Availability

The data presented in this study are available on request from the authors.

## Supplementary Materials

The following supporting information can be downloaded at: https://www.mdpi.com/article/10.3390/molecules30122625/s1, Supplement containing data.

## Abbreviations

SVMP	snake venom metalloproteinase
PLA2	phospholipase A2
DBK	dabsylated bradykinin
DBK1-7 (or 1-5, 1-8)	enzymatic fragment 1-7 (or 1-5, 1-8) of DBK
RBC	red blood cell
ACE	angiotensin-converting enzyme
CPN	carboxypeptidase N
NKA	Na^+^/K^+^-ATPase
PBS	phosphate-buffered saline
