# Supplementary material for: Strophanthus sarmentosus Extracts and the Strophanthus Cardenolide Ouabain Inhibit Snake Venom Proteases from Echis ocellatus
Source: Molecules. 2025 Jun 17;30(12):2625. doi: 10.3390/molecules30122625 (PMC12195819; doi:10.3390/molecules30122625)
Supplement: Supplementary file 1 [file molecules-30-02625-s001.zip › molecules-3608677-supplementary.pdf]

Supplement

Strophanthus sarmentosus extracts and the Strophanthus cardenolide ouabain inhibit snake venom proteases from *Echis ocellatus*

Julius Abiola [1,2], Olapeju Aiyelaagbe [2], Akindele Adeyi [3], Babafemi Ajisebiola [4], and Simone König [1*]

[1] IZKF Core Unit Proteomics, University of Münster, 48149 Münster, Germany; koenigs@uni-muenster.de (S.K.)

[2] Organic Unit, Department of Chemistry, University of Ibadan, Ibadan, Nigeria; abiolajulius005@gmail.com (J.A.), oaiyelaagbe@gmail1.com (O.A.)

[3] Animal Physiology Unit, Department of Zoology, University of Ibadan, Ibadan, Nigeria; delegenius@yahoo.com (A.A.)

[4] Department of Animal and Environmental Biology, Osun State University, Osogbo, Nigeria; dbabslinks@yahoo.com

* Correspondence: koenigs@uni-muenster.de; Ph.: 0049-251 8357164

Table S1: Anti-hemorrhagic activity of *S. sarmentosus* leaf and root extracts as expressed in the size of lesions on rat skin. Rats were injected with 0.2 mL of either saline, venom, venom + antivenom, or venom + extracts. Data are expressed as means ± StD of three individual experiments. The lesion area was estimated assuming an elliptic form (half of length * half of width * π).

	**Extract / mg/kg**	**Average lesion length / mm**				
			**Leaf**		**Root**	
			**Methanol**	**Ethylacetate**	**Methanol**	**Ethylacetate**
**Venom**		34±1				
**Antivenom**		17±7				
**Saline**		No foci				
**Venom**	**100**		23±2	32±1	37±5	27±2
	**200**		22±2	23±2	30±2	22±2
	**300**		25±9	15±2	22±1	17±2

	**Extract / mg/kg**	**Average lesion width / mm**				
			**Leaf**		**Root**	
			**Methanol**	**Ethylacetate**	**Methanol**	**Ethylacetate**
**Venom**		35±5				
**Antivenom**		15±3				
**Saline**		No foci				
**Venom**	**100**		25±2	23±2	35±3	17±2
	**200**		20±2	24±3	32±3	19±2
	**300**		35±5	12±2	22±1	10±2

	**Extract / mg/kg**	**Average lesion cross section / mm**				
			**Leaf**		**Root**	
			**Methanol**	**Ethylacetate**	**Methanol**	**Ethylacetate**
**Venom**		40±3				
**Antivenom**		16±3				
**Saline**		No foci				
**Venom**	**100**		24±2	25±2	38±2	20±2
	**200**		25±1	26±2	34±1	21±1
	**300**		40±3	13±1	22±2	15±3

	**Extract / mg/kg**	**Lesion area / mm^2**				
			**Leaf**		**Root**	
			**Methanol**	**Ethylacetate**	**Methanol**	**Ethylacetate**
**Venom**		943				
**Antivenom**		205				
**Saline**		No foci				
**Venom**	**100**		457	569	1026	363
	**200**		334	446	737	333
	**300**		687	148	380	141

Table S2: Effect of *S. sarmentosus* leaf and root extracts on venom hemolysis activity. Experiments were performed with mixtures of citrated red blood cells with either distilled water (control), venom, venom + antivenom, or venom + extracts. Data (absorption units) are expressed as means ± StD of three individual experiments.

	**Extract / mg/kg**	**Average hemolysis effect / au**				
			**Leaf**		**Root**	
			**Methanol**	**Ethylacetate**	**Methanol**	**Ethylacetate**
**Venom**		0.408±0.057				
**Antivenom**		0.055±0.015				
**Control**		2.892±0.008				
**Venom**	**100**		0.230±0.013	0.217±0.017	0.241±0.011	0.257±0.012
	**200**		0.184±0.011	0.127±0.005	0.191±0.008	0.151±0.010
	**300**		0.146±0.011	0.095±0.012	0.167±0.011	0.151±0.022

Table S3: Effect of *S. sarmentosus* leaf and root extracts on hemolysis activity of *venom* (IC_{50}, mg/mL).

**Extract**	**Methanol**	**Ethylacetate**
**Leaf**	161.90	104.45
**Root**	188.89	166.70

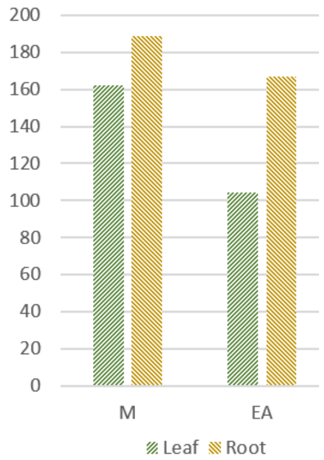

Table S4: Effect of ouabain on venom hemolysis activity. Experiments were performed using citrated red blood cells with of either distilled water (control), venom, venom + antivenom, or venom + ouabain. Data (absorption units and % inhibition) are expressed as means ± StD of three individual experiments. The experiments with pure ouabain were performed in a second laboratory as separate set of experiments using a different photometer compared to Tables S2/S3, which explains the slight differences in the measured values. IC_{50} = 3.13 mg/mL.

	**Concentrations/ mg/mL**	**Average hemolysis effect / au**	**% Inhibition**
**Venom**		0.406 ± 0.002	75.54
**Antivenom**		0.123 ± 0.002	69.85
**Control 100% hemolysis**		1.662 ± 0.007	
**Ouabain**	2.0	0.232 ± 0.013	42.77
	1.0	0.235 ± 0.009	41.973
	0.5	0.269 ± 0.017	33.53

Table S5: Influence of *S. sarmentosus* leaf and root extracts on coagulation time inflicted by venom. Experiments were performed with plasma adding either methanol root extract or ouabain (2 mg/mL) as controls, venom, venom + antivenom, venom + extracts or venom + ouabain (2 mg/mL).

	**Extract / mg/kg**	**Average coagulation time / s**				
			**Leaf**		**Root**	
			**Methanol**	**Ethylacetate**	**Methanol**	**Ethylacetate**
**Venom**		48.00				
**Antivenom**		85.67				
**Methanol root extract**		69.00				
**Venom**	**100**		240.00	119.00	228.00	99.00
	**200**		167.00	86.00	189.00	88.67
	**300**		123.00	77.30	179.00	84.00
**Ouabain**		69.00				
**Venom + ouabain**		116.00				
